# Heritability of Serum Apolipoprotein Concentrations in Middle-Aged Japanese Twins

**DOI:** 10.2188/jea.JE20081037

**Published:** 2009-09-05

**Authors:** Yang Ping Cai, Kazuo Hayakawa, Reiko Nishihara, Kenji Kato

**Affiliations:** 1 Department of Health Promotion Sciences, Graduate School of Medicine, Osaka University, Suita, Osaka, Japan; 2 Department of Nursing, International University of Health and Welfare, Odawara, Kanagawa, Japan

**Keywords:** apolipoprotein, heritability, adult twins

## Abstract

**Background:**

Studies of the genetic and environmental influences on apolipoproteins have been conducted, but few have used data from Japanese twins. The aim of this study was to quantify and compare the genetic and environmental causes of individual differences in the serum concentrations of apolipoproteins in Japanese middle-aged twins.

**Methods:**

Apo A-I, apo A-II, apo B, apo C-II, apo C-III, and apo E were studied. A total of 142 twin pairs, aged 45 through 65 years, were enrolled: 85 monozygotic pairs (59 male, 26 female) and 57 same-sexed dizygotic pairs (43 male, 14 female). The intraclass correlation coefficient and structural equation modeling were used to estimate the best-fitting model and heritability.

**Results:**

Sixteen percent to 75% of the total variances of apo A-I, apo C-II, and apo C-III were attributable to genetic influence; apo A-I and apo C-II were influenced by dominant genetic factors. Twenty percent to 73% of the total variances of apo A-II, apo B, and apo E were attributable to additive genetic influence; apo B was clearly influenced by common environmental factors. Furthermore, the heritability of all apolipoproteins was higher among females than among males.

**Conclusions:**

Genetic factors, including additive genetic effects (A) and dominant effects (D), influence apolipoprotein levels. However, a common environment does not influence the variances of these apolipoproteins, with the exception of apo B. Furthermore, the heritability of apolipoprotein phenotypes differs by sex.

## INTRODUCTION

Apolipoproteins are proteins that bind to fats (lipids) to form a lipoprotein. They are synthesized in the liver and intestine and form lipoproteins that transport dietary fats through the bloodstream. Apolipoproteins also serve as enzyme cofactors, receptor ligands, and lipid transfer carriers that regulate the metabolism of lipoproteins and their uptake in tissues. There are 6 major classes of apolipoproteins, and several sub-classes. These include A (apo A-I, apo A-II, apo A-IV, and apo A-V), B (apo B48 and apo B100), C (apo C-I, apo C-II, apo C-III, and apo C-IV), D, E, and H. In the present study, A (apo A-I and apo A-II), B (apo B48 and apo B100), C (apo C-II and apo C-III), and E were studied.

Apolipoprotein A-I is the most abundant protein in high-density lipoproteins (HDL), and there is an inverse relation between both HDL-cholesterol and plasma apo A-I levels and the risk of coronary heart disease (CHD).

In humans, apo A-II is the second most abundant apolipoprotein in HDL. It has been hypothesized that apo A-II is not a strong determinant of lipid metabolism, but that it is instead a modulator of reverse cholesterol transport.^1^

Apolipoprotein C-II is found in chylomicrons (large lipoprotein particles absorbed in the gastrointestinal tract) and very low-density lipoprotein (VLDL: large lipoproteins that are broken down to eventually form low-density lipoprotein). Apo C-II activates the enzyme that breaks down chylomicrons and VLDL molecules.

Apolipoprotein C-III is a VLDL protein that inhibits lipoprotein lipase and hepatic lipase, and is believed to delay catabolism of triglyceride-rich particles. An increase in apo C-III level induces hypertriglyceridemia.

Apolipoprotein B is a form of low density lipoprotein (LDL), and plays a role in metabolism.

Apolipoprotein E is a serum protein that is involved in the transport, storage, and metabolism of cholesterol. Apo E was first recognized for its importance in lipoprotein metabolism and cardiovascular disease.^2^ More recently, investigators have focused on its role in several biological processes not directly related to lipoprotein transport, including Alzheimer's disease (AD), immunoregulation, and cognition.^3^^–^^6^

In Japan, the twin birth rate is approximately 4 twin pairs per 1000 newborn infants. Twin studies are valuable because monozygotic (MZ) twins share an identical genotype, whereas dizygotic (DZ) twins are no more alike genetically than siblings, who share an average of 50% of their segregating genes. If MZ twins display a greater resemblance for a specific trait than do DZ twins, it is likely to be attributable to genetic factors. Although there have been studies of the genetic and environmental influences on apolipoproteins,^7^^–^^9^ few have analyzed Japanese twins. Therefore, the aim of this study was to quantify and compare the genetic and environmental causes of individual differences in serum apolipoprotein levels in Japanese middle-aged twins.

## METHODS

### Subjects

The twins participating in this study were recruited from the cohort of the nationwide, population-based, Osaka University Aged Twin Registry (OUATR) in Japan. Twin pairs in the OUATR were recruited in several ways, including newspaper advertisements, posters in hospitals, and referrals from midwives. A total of 188 same-sexed pairs volunteered for this study and underwent a comprehensive medical examination. The ages of the twins varied from 12 to 90 years; most were in their sixth and seventh decades of life. A total of 142 twin pairs aged from 45 through 65 years were chosen as the subjects for this study, including 85 MZ pairs (59 male, 26 female) and 57 same-sexed DZ pairs (43 male, 14 female), as shown in Table 1.

**Table 1.** Distribution of the Respondents by Sex and Zygosity

	No. of pairs (%)		Total
	
	MZ	DZ	
Male	59 (42%)	43 (30%)	102 (72%)
Female	26 (18%)	14 (10%)	40 (28%)
Total	85 (60%)	57 (40%)	142 (100%)

Abbreviations: DZ, dizygotic twins; MZ, monozygotic twins.

This study was approved by the Osaka University Ethical Review Committee.

### Zygosity

When the twin registry was initially compiled, beginning in 1974, zygosity was determined by using the phenylthiocarbamide (PTC) test and tests of 9 blood systems: ABO, Rh (C, c D, E, e), MN (M, N), Lewis (Lea, Leb), P (P1), Duffy (Fya, Fyb), Kidd (JKa, JKb), Kell (K), and Diego (Dia). This classification methodology is 95% accurate in determining twin zygosity. The use of a questionnaire is also 95% accurate in determining zygosity. Such a questionnaire was used in a study of a Swedish cohort.^10^ Thus, the zygosity status of our source population was determined with an overall accuracy of 95%.

### Method

The twins were instructed in advance not to eat breakfast on the examination day. Ten ml venous blood was taken at the Kinki University Hospital. Apolipoproteins were analyzed by single radial immunodiffusion. All blood analyses were done at the Central Laboratory of Kinki University Hospital in Japan.

Intraclass correlation coefficient (ICC) and structural equation modeling were used to estimate the best-fitting model and heritability.^11^

Twin models, including additive genetic effects (A), dominant effects (D), common environmental effects (C), and non-shared environmental effects (E), were examined using the standard correlations between MZ and DZ pairs, ie, the correlation of additive genetic effect was set to 1 for MZ pairs and to 0.5 for DZ pairs and the correlation of dominant genetic effect was set to 1 for MZ pairs and to 0.25 for DZ pairs. The correlation between common environmental effects equaled 1; there is no correlation of nonshared environmental effects between twins. It is assumed that common environmental factors are shared to a similar extent by MZ and DZ pairs. If the MZ twin correlation is more than double the DZ twin correlation, it is possible that dominant genetic effects played a large role in that trait. Because some ICCs of MZ pairs were more than double those of DZ pairs in this study (Table 3), we fit the full model ACE and ADE. Akaike’s information criterion (AIC) and the chi-square test were used to evaluate the goodness of fit of the models.

SPSS 13.0 for Windows was used for the statistical analysis. Genetic and environmental sources of variation in the serum levels of apolipoprotein were modeled by MxGui Version 1.7.03.

## RESULTS

### Descriptive Analysis

The means and standard deviations of serum apolipoprotein levels are shown in Table 2. In a comparison of men and women, a significant (*P* < 0.05) difference was noted in the levels of all apolipoproteins except apo A-II and apo C-II; all apolipoprotein levels were higher in women than in men. Therefore, subsequent analysis examined differences by sex. Most of the mean apolipoprotein values in this study were higher than the average normal values, but only the apo B value among women exceeded the normal range. We attribute this finding to sampling error.

**Table 2.** Mean Serum Concentrations of Apolipoproteins, by Sex

	Males		Females		*P*^a^
		
	Mean	S.E.	Mean	S.E.	
Apo A-I (mg/dl)	130.06	27.44	139.66	27.25	0.007
Apo A-II (mg/dl)	32.49	6.55	33.58	6.96	0.207
Apo C-II (mg/dl)	3.68	1.74	3.89	1.48	0.334
Apo C-III (mg/dl)	8.57	3.29	9.84	3.37	0.004
Apo B (mg/dl)	95.16	23.70	108.16	35.08	0.001
Apo E (mg/dl)	4.16	1.25	5.01	1.52	0.000

^a^The paired samples *t* test was used for the analysis.

### Twin correlations

Table 3 shows the intraclass correlation coefficients for apolipoprotein values in male and female MZ and DZ pairs. Although all the intraclass correlation coefficients for MZ pairs were high, and higher than those for DZ pairs, there was no significant difference in ICC between MZ and DZ pairs, in either men or women (*P* > 0.05, by z test).

**Table 3.** Model-fitting Results and Intraclass Correlation Coefficients

			Standardized variances							Correlations		
				
			a^2^	c^2^ or d^2^	e^2^	χ^2^	df	*P*	AIC	rMZ	rDZ	*P*^a^
Apo	Male	ACE	0.37 (0.00, 0.66)	0.00 (0.00, 0.46)	0.63 (0.44, 0.90)	1.63	3	0.65	−4.36	0.39	0.06	0.84
AI		ADE	0.00 (0.00, 0.64)	0.39 (0.00, 0.67)	0.61 (0.43, 0.88)	1.31	3	0.75	−4.78			
	Female	ACE	0.60 (0.39, 0.88)	0.00 (0.00, 0.40)	0.40 (0.27, 0.63)	6.00	3	0.11	0.01	0.61	0.18	0.88
		ADE	0.30 (0.11, 0.58)	0.30 (0.10, 0.66)	0.40 (0.26, 0.62)	5.94	3	0.11	−0.05			
Apo	Male	ACE	0.30 (0.00, 0.44)	0.05 (0.00, 0.31)	0.65 (0.39, 0.86)	2.46	3	0.48	−3.53	0.31	0.22	0.38
AII		ADE	0.35 (0.09, 0.64)	0.00 (0.00, 0.33)	0.65 (0.46, 0.94)	21.34	3	0.00	15.34			
	Female	ACE	0.65 (0.34, 0.89)	0.00 (0.00, 0.41)	0.35 (0.00, 0.59)	2.48	3	0.47	−3.51	0.56	0.26	0.69
		ADE	0.12 (0.00, 0.50)	0.55 (0.10, 0.89)	0.33 (0.22, 0.54)	20.72	3	0.00	14.72			
Apo	Male	ACE	0.61 (0.18, 0.93)	0.00 (0.00, 0.36)	0.39 (0.28, 0.59)	10.87	3	0.01	4.87	0.56	0.21	0.25
CII		ADE	0.10 (0.00, 0.48)	0.52 (0.30, 0.72)	0.38 (0.27, 0.56)	4.21	3	0.24	−1.79			
	Female	ACE	0.75 (0.21, 1.00)	0.00 (0.00, 0.39)	0.25 (0.17, 0.39)	11.51	3	0.01	5.51	0.77	0.05	0.56
		ADE	0.00 (0.00, 0.38)	0.75 (0.48, 0.91)	0.25 (0.16, 0.38)	4.92	3	0.18	−1.07			
Apo	Male	ACE	0.00 (0.00, 0.33)	0.14 (0.00, 0.46)	0.86 (0.53, 1.00)	30.22	3	0.00	24.22	0.56	0.13	0.56
CIII		ADE	0.16 (0.01, 0.44)	0.00 (0.00, 0.43)	0.84 (0.52, 0.98)	1.92	3	0.58	−4.07			
	Female	ACE	0.00 (0.00, 0.38)	0.71 (0.37, 0.99)	0.29 (0.20, 0.42)	24.61	3	0.00	18.61	0.71	0.27	0.71
		ADE	0.67 (0.35, 0.93)	0.00 (0.00, 0.31)	0.33 (0.23, 0.50)	1.83	3	0.60	−4.17			
Apo	Male	ACE	0.32 (0.05, 0.67)	0.29 (0.00, 0.56)	0.39 (0.28, 0.55)	6.35	3	0.09	0.35	0.66	0.25	0.34
B		ADE	0.62 (0.30, 0.90)	0.00 (0.00, 0.34)	0.38 (0.28, 0.54)	6.72	3	0.08	0.72			
	Female	ACE	0.59 (0.20, 0.90)	0.16 (0.00, 0.53)	0.25 (0.17, 0.40)	1.29	3	0.73	−4.71	0.74	0.47	0.84
		ADE	0.75 (0.24, 0.99)	0.00 (0.00, 0.32)	0.25 (0.16, 0.39)	1.44	3	0.69	−4.56			
Apo	Male	ACE	0.48 (0.17, 0.79)	0.00 (0.00, 0.24)	0.52 (0.37, 0.77)	6.42	3	0.09	0.41	0.43	0.21	0.40
E		ADE	0.20 (0.06, 0.68)	0.30 (0.09, 0.71)	0.50 (0.35, 0.75)	6.64	3	0.08	0.64			
	Female	ACE	0.73 (0.41, 0.97)	0.00 (0.00, 0.29)	0.27 (0.18, 0.40)	5.80	3	0.12	−0.19	0.77	0.49	0.95
		ADE	0.01 (0.00, 0.57)	0.73 (0.43, 0.99)	0.26 (0.17, 0.39)	8.44	3	0.04	2.44			

Figures in parentheses are 95% confidence intervals.

^a^The z test was used for the analysis.

### Results of model fitting

Structural equation modeling was used to estimate heritability. Table 3 shows the fit statistics for the full model, including parameter estimates and their 95% confidence intervals. The full (ACE or ADE) model adequately fit the data. When the correlation for DZ twins was less than half that for MZ twins, it is the result of a dominant genetic trait. However, in the present study, not all ADE models were better than the ACE model when ICCs in MZ pairs were twice those of DZ pairs. We chose the models for which the Akaike information criterion value was lower and *P* was higher than 0.05 by the chi-square test. As the results in Table 3 show, apo A-I, apo C-II, and apo C-III were better fitted to the ADE model, and the total variance of apolipoproteins that was attributable to genetic influence ranged from 16% to 75%. Apo A-I and apo C-II were influenced by dominant genetic factors. Apo A-II, apo B, and apo E were better fitted to the ACE model, and the total variance of apolipoproteins that was attributable to additive genetic influence ranged from 20% to 73%. Apo B was clearly influenced by common environmental factors. Finally, the heritability of all apolipoproteins was greater among women than among men.

## DISCUSSION

The present study investigated the magnitude of genetic and environmental effects on serum levels of apolipoprotein in 142 middle-aged Japanese twin pairs. The importance of the influence of genetic factors on a trait is defined as heritability, ie, the proportion of population variation attributable to genetic variation, including additive genetic effects (A) and dominant effects (D). We found that 16% to 62% and 59% to 75% of the total variances were attributable to genetic factors in men and women, respectively.

Regarding the variance in apolipoprotein A-I level, 39% and 60% was attributable to genetic factors in men and women, respectively. Most studies report heritability estimates between 40% and 80% for apo A-I and apo B, which conforms to our results.^9^^,^^12^^,^^13^ However, some investigators found no evidence of additive genetic effects.^14^^,^^15^ In addition, it was reported that variations in apo A-I levels that are within the normal range, especially during adolescence, are likely to be influenced by many factors, without any significant contribution from major genes.^16^

Concerning apolipoprotein B, 32% and 59% of variance was due to genetic factors in men and women, respectively. This accords with some other studies,^9^^,^^13^^,^^17^ which reported that the heritability of apo B ranged from 28% to 78%. Chien et al reported that, among adolescents, variations in apo B levels that are within the normal range are controlled by a major gene.^18^ In addition, apo B was found to be significantly affected by occupation, alcohol consumption, physical activity, and obesity.^19^ Apo B is strongly influenced by lifestyle.

The heritability of apo E in the present study was 50% and 74% in men and women, respectively. Beekman et al reported that the total variance in apolipoprotein E levels depended largely on genetic factors (from 48% to 87%),^20^ which conforms with the results of the few previous reports.^14^^,^^21^

There are fewer studies of the heritability of apolipoprotein A-II, C-II, and C-III. Hamsten et al reported that the genetic heritability of serum apolipoprotein A-II was 42%, as estimated by path analysis in families selected through probands with premature myocardial infarction and in families randomly selected from the general population.^22^ This estimate accords with our finding that the genetic heritability of serum apolipoprotein A-II was 30% for male twins and 65% for female twins. There are very few studies of the heritability of apo C-II and C-III.^19^ In the present study, we were unable to obtain exact estimates of the genetic effects on the variance in apo C-II and C-III. We believe that this is the first report of these values: the heritability of apo C-II is 62% in men and 75% in females and the heritability of apo C-III is 16% in males and 67% in females.

When the correlation among DZ twins was less than half the correlation among MZ twins, models including a dominance genetic factor were examined. In the present study, the influence of dominant genetic factors on apo A-I was 39% in males and 30% in females; the influence on apo C-II was 52% in males and 75% in females. However, Beekman reported^19^ that none of these models fitted significantly better than a model that included only an additive genetic and unique environmental factor. Therefore, further twin studies of the dominant genetic factors that influence apolipoprotein should prove informative.

In previous studies, the estimates of the age and sex factors that influence the heritability of apolipoprotein are contradictory, but most results indicate that age and sex play important roles in the heritability of apolipoprotein. For example, in a study that used data from the Swedish Twin Registry^23^ to evaluate the effect of aging on genetic and environmental variation in lipid and apolipoprotein levels, increases in age-related phenotypic variance in total cholesterol and apo B were almost entirely due to the accumulation of unique environmental experiences; no consistent age trends were found for apo A-I. The limited number of available longitudinal studies provide evidence for the existence of age-dependent gene expression for at least some lipids and lipoproteins. New genes are expressed during adolescence,^24^ and gene expression differs in childhood and adulthood.^25^ There is a sex-specific influence on variation in apo B levels,^18^ and a small, but significant, sex difference in heritability of lipoprotein A was observed.^26^ However, Debra et al reported that there are no sex differences in the relative importance of genetic factors for any of the lipids studied, except apolipoprotein B.^27^

In the present study, because twin pairs aged younger than 45 or older than 65 years were few—and age significantly influences heritability of apolipoprotein—twin pairs aged from 45 through 65 years were selected as subjects. Among these middle-aged twin pairs, sex differences in the genetic factors influencing apolipoprotein were observed: both heritability and the levels of apolipoprotein were higher in females than in males. We hypothesize that new genes in apolipoprotein are expressed during middle age in women,^28^ and that adverse changes in apolipoproteins occur after menopause in women.^13^ Furthermore, in Japan, middle-aged men usually work, so their environments and lifestyles may differ considerably from each other. By contrast, most women do housework and their living environments and lifestyles may be more similar. Therefore, environmental influences may be stronger among men as compared to women.

Shared environmental factors are usually regarded as those related to early childhood. Some studies have shown that common environmental factors significantly influenced apolipoprotein traits.^13^^,^^29^^–^^32^ The importance of shared environmental influences on apolipoproteins during early adulthood suggests that family-based lifestyle interventions could be successful.^33^ However, in the present study, common environmental factors influenced only apo B. This result accords with a previous study of 1362 twin pairs that concluded that none of the lipid traits was significantly influenced by the presence of a shared environment.^27^

The present study did have some limitations. The number of subjects was not large; thus, the 95% confidence intervals were large and the statistical power relatively low. However, we believe that data from 142 twin pairs is not inconsiderable, and should have substantial value as a reference.

### Conclusion

In conclusion, we found that genetic factors, including additive genetic effects (A) and dominant effects (D), influenced apolipoprotein levels, and that a common environment does not influence the variances of apolipoproteins, with the exception of apo B. Furthermore, the heritability of these apolipoprotein phenotypes differs by sex.
